# Carbohydrate Mouth Rinse Effects on Exercise Capacity in Pre- and Postprandial States

**DOI:** 10.1155/2011/385962

**Published:** 2011-07-27

**Authors:** Elie-J. M. Fares, Bengt Kayser

**Affiliations:** ^1^Institute of Movement Science and Sports Medicine, Faculty of Medicine, University of Geneva, 1211 Geneva 4, Switzerland; ^2^Institute of Literature and Human Sciences, Physical Education Department, University of Balamand, Beirut, Lebanon

## Abstract

*Background*. Oropharyngeal receptors signal presence of carbohydrate to the brain. Mouth rinses with a carbohydrate solution facilitate corticomotor output and improve time-trial performance in well-trained subjects in a fasted state. We tested for this effect in nonathletic subjects in fasted and nonfasted state. *Methods*. 13 healthy non-athletic males performed 5 tests on a cycle ergometer. After measuring maximum power output (Wmax), the subjects cycled four times at 60% Wmax until exhaustion while rinsing their mouth every 5 minutes with either a 6.4% maltodextrin solution or water, one time after an overnight fast and another after a carbohydrate rich breakfast. *Results*. Mouth rinsing with maltodextrin improved time-to-exhaustion in pre- and postprandial states. This was accompanied by reductions in the average and maximal rates of perceived exertion but no change in average or maximal heart rate was observed. *Conclusions*. Carbohydrate mouth rinsing improves endurance capacity in both fed and fasted states in non-athletic subjects.

## 1. Background

As the primary fuel for abiding sports, carbohydrate intake before, during, and after exercise, has a positive effect on endurance performance [1–6]. Apart from being an energy substrate, the sight, smell, and taste of food may act as positive reinforcements. By generating promises for food intake, these senses play a role in reward prediction. As a result, the body starts to function as if it is going to receive food [7–10]. Recent studies suggest that simple mouth rinses with a carbohydrate solution can improve endurance performance even for performances that last about an hour, that is, in conditions where glycogen stores should not be limiting [11–15]. Both complex and simple sugars can elicit a mouth rinse effect [11–14]. Intravenous infusion of glucose with a similar effect on blood sugar regulation parameters, as compared to a swallowed glucose solution, did not affect performance in a ~1-hour-time trial. It seems that specific oropharyngeal receptors, linked to brain centers that are involved in motivation and reward, play a role in the ergogenic effect of carbohydrate mouth rinsing [16]. The first experiments were done on cycle ergometers and involved young well-trained subjects who were asked to finish a specific amount of work in a minimum of time (time trial). When rinsing their mouths with a nonsweet carbohydrate solution subjects were faster as compared to rinsing with water [12–14]. 

Not all subsequent mouth-rinse protocols successfully increased endurance performance, as some studies showed a lack of enhancement of time-trial performance in the fed state on a cycle ergometer [17] while another study reported no improvement when using a treadmill [18]. 

All those studies involved well-trained subjects and used similar time-trial experimental paradigms. Given the rather surprising nature of the potential underlying mechanisms for these findings and the need to further probe the hypothesis of exercise enhancement through oropharyngeal receptor stimulation, we therefore decided to extend the existing evidence base by investigating the effect of an oral carbohydrate stimulus on exercise capacity using a time-to-exhaustion test, comparing fed and fasted states. And since no other studies tested this effect in a nonathletic population, we hypothesized that a carbohydrate mouth rinse would affect endurance capacity in a nonathletic population and would have more effect in the fasted state as compared to the fed state.

## 2. Methods

### 2.1. Subjects

We recruited 13 nonathletic male subjects to participate in this study. The study was approved by the ethical committee of the University of Balamand in Beirut, Lebanon. Subjects signed an informed consent form and could withdraw at any moment from the study. Exclusion criteria were a sedentary lifestyle, active training for endurance sports, and any preexisting illness.

### 2.2. Experimental Design

Tests were done in an exercise physiology laboratory at the University of Balamand. Room temperature was maintained at 20 degrees centigrade during experiments. Humidity varied between 52 and 56%. A fan was set 2 m away from the participants to provide cooling during exercise. Subjects were asked to refrain from any strenuous activity, alcohol, caffeine, or any other form of stimulant during the last 24 hours preceding tests. Each participant did 5 tests on a cycle ergometer (Monark, Ergomedic 839E, used in power setting, rpm independent). On their first visit, the participants did an incremental test to exhaustion, based on Kuiper's protocol [14, 19]. After a 5 min warm-up at 100 Watts, the power was increased 50 Watts every 2.5 min until the heart rate reached 160 bpm. Power was then increased 25 Watts every 2.5 minutes until exhaustion. Maximal workload (Wmax) was then calculated as Wout + (*t*/150) × 25, where Wout refers to the last completed stage and *t* to the time of the unfinished stage [14]. On separate days, the subjects then performed 4 time-to-exhaustion tests. Time between tests was not less than 72 hrs and not more than 96 hrs. After 5 min warm-up at 50 Watts, power was set at 60%Wmax and subjects would cycle until exhaustion. Based on preliminary experiments in subjects of similar training status, we chose 60%Wmax since higher percentages yielded exercise times that were too short. Subjects were required to keep the pedaling rate between 70 and 100 rpm; when they failed to pedal at least at 70 rpm, the test ended. Heart rate was monitored continuously with telemetry (Nike Triax C5, NY, USA) and subjects were asked to rate on a 10 point Borg scale [20, 21] every 5 minutes, just after each mouth rinsing, their rate of perceived exertion (RPE). 

On 2 occasions, subjects would rinse their mouth with a 25 mL maltodextrin solution (6.4%, CHO) and on the 2 other with water (PLA) for 5–10 seconds every 5 min. Subjects were not allowed to swallow the solution and had to spit it out; they were however allowed to drink water from a separate bottle *ad libitum*. For both solutions, subjects would once start the test after an overnight fast (FCHO and FPLA) and once after a standardized carbohydrate-rich breakfast (CHO and PLA). Breakfast included sandwiches/cereals/oatmeal along with fat-free milk/fruit juice (no coffee or tea), fresh fruits (bananas/figs), and dried fruits (raisins/apricots/dates). Breakfast was taken 3 hours before the test. Tests were done between 09 h 30 and 11 h 30 AM and subjects did the paired (fasted versus fed) trials at the same time of the day. After each test, the subjects were asked to indicate what solution they thought they had received. They could listen to music of their choice during the test and were able to see the time elapsed. Heart rate was not given to them. During the tests, the researcher and the participant were the only two people inside the laboratory. The order of tests was done in a randomized balanced order and the subjects were blinded to the mouth-rinse solution.

### 2.3. Data Analysis

Data were analyzed using PASW statistics (version 18.0.0, Chicago, Ill, USA). Data are reported as means ± standard deviation. Effects of mouth rinsing with maltodextrin and prandial state on time-to-exhaustion, heart rate, and RPE were analysed using a repeated measures two-by-two way ANOVA. Sphericity was tested for with Mauchly's test. Solution identification values were analysed using Chi-square analysis. The level of significance was set at *P* < 0.05.

## 3. Results 

### 3.1. Subjects

Average age (±SD) was 21 ± 3 yrs, body mass 83 ± 8 kg, stature 180 ± 6 cm. Maximum exercise capacity was 200 ± 18 Watt corresponding to an estimated maximum aerobic capacity of 31 ± 7 mL O_2_/kg/min or 8.9 ± 2.0 METs. Resting heart rate was 72 ± 6 bpm, maximum heart rate was 167 ± 10 bpm, 75 ± 4% of expected (Karvonen). Subjects did the time-to-exhaustion trials at 120 ± 11 Watt (60% Wmax).

### 3.2. Time-to-Exhaustion, Heart Rate, and RPE

**Figure 1:**
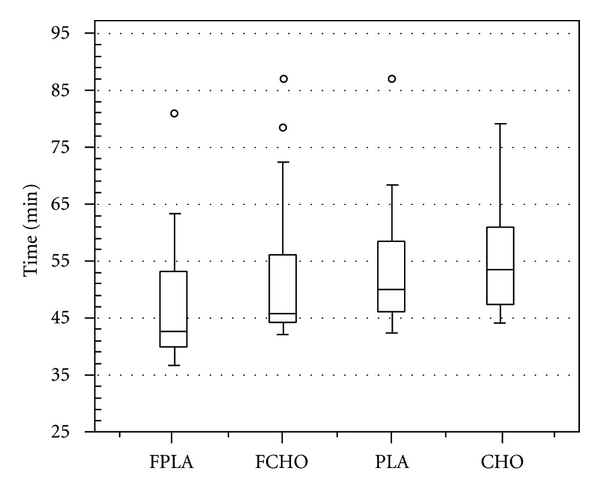
Time-to-exhaustion. Boxplot of the time-to-exhaustion for the 4 different trials (FCHO: carbohydrate rinse in fasted state; FPLA: placebo in fasted state; CHO: carbohydrate rinse in fed state; PLA: placebo in fed state). The thick line in the boxes represents the median, the extremes of the boxes the 25 and 75% percentiles, and the whiskers the minimum and maximum values. Outliers are indicated by a circle when more than 1.5 times the interquartile ranges beyond the 75% quartile.

Average values for time-to-exhaustion, mean exercise heart rate, maximum exercise heart rate, mean exercise RPE, and maximum RPE are shown in Table 1. The repeated measures two-by-two ANOVA revealed a main effect of maltodextrin mouth rinsing on time-to-exhaustion (*P* = 0.020), no effect of prandial state (*P* = 0.220) and a tendency for interaction (*P* = 0.075) (see also Figure 1). Heart rate increased with time in a similar fashion for each trial. There was no main effect of mouth rinsing or prandial state on mean exercise heart rate but a significant interaction (*P* < 0.0001). The same was found for maximum heart rate (*P* < 0.0001). RPE also increased throughout each test. There was a significant main effect of mouth rinsing on average exercise RPE (*P* = 0.032) but not of prandial state. There was no interaction. Similar results were found for maximum RPE with a main effect of mouth rinsing (*P* = 0.035) and no effect of prandial state or interaction.

### 3.3. Solution Identification

For the FCHO trial, 8 subjects out of 13 identified the correct solution, for the FPLA trial 9 subjects out of 13. For both the PLA and CHO trials, 6 out of 13 identified the correct solution. Chi-square was 0.692 (*P* = 0.405), preventing the rejecting of the null-hypothesis of 50-50% chance to guess correctly.

## 4. Discussion

The main finding was that a mouth rinse with a maltodextrin solution improved time-to-exhaustion in both pre- and postprandial states by an average 7 ± 3% and 3 ± 2%, respectively. This improvement was accompanied by an average 6 ± 1% drop in the level of perceived exertion when compared to placebo. The ergogenic effect of carbohydrate presence in the mouth tended to be more evident on an empty stomach. Our results complement those reported in earlier studies [12–15], by adding time-to-exhaustion on a cycle ergometer, as another exercise performance testing paradigm in which oral carbohydrate rinsing, has an effect and by reporting that this effect is present in young and apparently healthy but nonathletic subjects.

We chose to use the original solutions used by Carter et al. [12] in their first study, a watery nonsweet tasting maltodextrin solution and just plain water as placebo. Saliva contains amylase, which breaks down starch into low-molecular-weight maltodextrins, but very few glucose units. There may have been some release of single glucose units while rinsing with the maltodextrin solution, which is probably what was needed in order to activate the purported CHO receptors in the oropharynx. Since the subjects were not allowed to swallow (and did not swallow) the rinse solution, very little carbohydrate made its way to the stomach and the intestinal system. We are not aware of any glucose transporters in the oropharynx, so there will be very little uptake of glucose in the condition of mouth rinsing with the maltodextrin solution. It cannot be totally excluded that some subjects had an idea of what they were taking since they indicated slight differences in texture for the two solutions, and in the fasted state, 65% of the subjects correctly distinguished between them. Also for practical reasons, the study was single-blinded, leaving potential for experimenter bias. But since in the fed state only 46% guessed correctly, with an overall (fed and fasted together) 56% correct guess not significantly different from pure chance (50%), placebo effects would rather seem unlikely. The intervention showed an interaction effect for average and maximum heart rate. This means that depending on prandial state the effect of the rinsing solution varied. The interpretation of this finding is not straightforward and can be a chance finding and should probably only be interpreted as an absence of a main effect. 

### 4.1. Other Studies

Carter et al. [12] were the first to report an effect of carbohydrate mouth rinsing solution on time-trial performance lasting ~1 hour. They came to their original experimental design from the observation that an oral glucose solution improved a ~1 hr cycling-time trial, while intravenous glucose administration did not [16]. Since then, several other studies from the same and from other laboratories were published, but not always reporting increased performance (see Table 2). Most studies that found an effect were carried out in the fasted state. Beelen et al. [17] looked specifically at the effects of carbohydrate mouth rinsing in the fed state and did not find any difference with placebo. They suggested that oral perception of carbohydrates perhaps only plays a role when muscle and liver glycogen stores are reduced [17]. Our findings would suggest that also in postprandial state the effect might occur, at least in our experimental settings.

 To explain their original findings, Carter et al. [12] mentioned a parasympathetic reflex triggered by the taste, smell, and sight of food and an associated cephalic phase of insulin release (CPIR), a sudden rise in insulin concentrations within minutes following gustatory stimulation resulting from a rapid mobilization of previously stored insulin in beta cells. This causes a small increase (~5 *μ*U/mL) in arterial plasma insulin [22] which would improve glucose uptake and maintain carbohydrate oxidation rates [12]. This cephalic phase of insulin release is well described for the presence of sweetness in the oral cavity [23] but has not been described for nonsweet-tasting carbohydrate such as used in the original experiment of Carter et al. [12] or our setup and remains to be investigated. Chambers et al. [13] combined a time-trial setup with fMRI experiments looking for the brain areas associated with on one hand oral presence of carbohydrate and on the other hand the presence of sweetness. Apart from confirming the performance enhancing effect of mouth rinsing with a carbohydrate solution, the authors found in a separate experimental setup that sweetness and carbohydrate sensing did not activate the same brain areas on fMRI and that carbohydrate sensing passed through the primary taste cortex and the putative secondary taste cortex and then activated brain areas involved in reward and motor control including the anterior cingulate cortex (ACC), parts of the basal ganglia (right caudate), and the ventral striatum [13]. It would thus seem that the performance enhancing effect of the presence of CHO in the oropharynx is related not to sweetness but to some other quality. This contention is corroborated by the recent findings of Gant et al. [24] who used an artificial sweetener in both the placebo and the maltodextrin solution and showed only an ergogenic effect for the latter. They reported that oral presence of carbohydrate facilitates corticomotor output to activated muscle not only in the fatigued state but also in the nonfatigued state and concluded that this type of sensorimotor integration probably also occurs without severe exercise-induced metabolic perturbations [24].

### 4.2. Perception

The oropharynx contains many receptors, including taste receptors distributed on the surface of the tongue, epiglottis, palate, and oesophagus, recognizing sweet, salt, bitter, sour, and umami tastes [25], and other kinds of oral somatosensory receptors, identifying viscosity, fat texture and temperature [9]. These receptors are now complemented with hitherto unknown oropharyngeal receptors that apparently sense the presence of complex and simple sugars, independently from sweetness.

Since food intake is indispensable for survival, it elicits a strong affective response, and in case of good palatability, a hedonic experience. This hedonic experience is referred to as “qualia” or “the hard problem of consciousness” because it is so difficult to investigate the relationship between physical phenomena, such as brain processes, and subjective experience [8, 10]. Also competitions, trophies, sport media, fame, and fortune represent positive reinforcement creating appetitive motivation. This appetitive motivation explains behavioural actions toward certain goals. It is linked to positive hedonic experiences from food, sex, and winning [26]. Mouth rinse carbohydrate solutions can apparently activate this appetitive motivation in order to increase endurance performance [12–14], force production [24], or endurance capacity (this study). The observation that this effect is perhaps more pronounced in the fasted state suggests that these receptors signal the CNS that exercise-related energy expenditure is possible without jeopardizing the organism's integrity. There is also evidence from animal studies of the presence of carbohydrate receptors independent of sweetness sensation. There are two types of sweetness taste receptors cells T1R2 and T1R3. These are presented as heterodimers and serve as natural-sugar and artificial-sweeteners detectors. T1R3 knockout mice were able to identify sucrose solutions. This suggests that the presence of T1R3-independent detection pathways expressed in homodimers like T1R2 or other non-T1R receptors [15, 27–29]. Despite these advances in animal and human research the brain circuitry involved in these regulatory mechanisms is not fully understood yet.

## 5. Conclusions

The human oropharynx is equipped with receptors that can signal the presence of simple and complex carbohydrate. In ~1 hr duration maximum endurance exercise (i.e., before depletion of endogenous carbohydrate stores), carbohydrate mouth rinsing significantly improves performance, especially in the fasted state. Our results show that this effect is also present in time-to-exhaustion type effort in young but nonathletic subjects.

## Figures and Tables

**Table 1:** Average values for time-to-exhaustion, heart rate, and RPE in the four conditions.

	Time-to-exhaustion	Mean HR	Max HR	Mean RPE	Max RPE
	min	min^−1^	min^−1^	a.u.	a.u.
CHO	56.6 ± 12.2	152 ± 7	164 ± 9	5.0 ± 0.7	8.6 ± 0.5
PLA	54.7 ± 11.3	159 ± 8	172 ± 9	5.5 ± 0.7	8.9 ± 0.4
FCHO	53.9 ± 12.8	155 ± 10	166 ± 12	5.2 ± 0.9	8.4 ± 1.0
FPLA	48.3 ± 15.3	152 ± 9	165 ± 9	5.4 ± 0.8	8.9 ± 0.2
ANOVA main effect of mouth rinse	F(1,12) = 7.212 *P* = 0.020	F(1,12) = 2.049 *P* = 0.178	F(1,12) = 2.962 *P* = 0.111	F(1,12) = 5.864 *P* = 0.032	F(1,12) = 5.660 *P* = 0.035
ANOVA main effect of prandial state	F(1,12) = 1.676 *P* = 0.220	F(1,12) = 0.983 *P* = 0.341	F(1,12) = 0.547 *P* = 0.474	F(1,12) = 0.237 *P* = 0.635	F(1,12) = 1.000 *P* = 0.337
Interaction	F(1,12) = 3.786 *P* = 0.075	F(1,12) = 42.646 *P* < 0.0001	F(1,12) = 27.351 *P* < 0.0001	F(1,12) = 1.500 *P* = 0.244	F(1,12) = 1.371 *P* = 0.264

a.u.: arbitrary units.

FCHO: carbohydrate rinse in fasted state; FPLA: placebo in fasted state; CHO: carbohydrate rinse in fed state; PLA: placebo in fed state; HR: heart rate; RPE: rate of perceived exertion.

Data are reported as means ± standard deviation.

**Table 2:** Summary of studies investigating the effects of a carbohydrate mouth rinse solution on high intensity exercise.

Paper	*n*	Subjects	Intensity	CHO	Performance measurement	Enhanced Endurance/performance	Times/Distance
Carter et al. [12, 16]	9	t	75% VO2max	6.4% MD versus PLA (water)	Time trial ~60 min (914 kJ ± 40 kJ)	Yes	59.57 ± 1.50 min versus 61.37 ± 1.56 min
Pottier et al. [14]	12	t	75% VO2max	CES (Gatorade) versus PLA (aspartame)	Time trial ~60 min (975 ± 85 kJ)	Yes	61.7 ± 5.1 min versus 64.1 ± 6.5 min
Chambers et al. [13] Part 1	8	t	75% VO2max	6.4% GLU versus PLA (saccharin)	Time trial ~60 min (914 ± 29 kJ)	Yes	60.4 ± 3.7 min versus 61.6 ± 3.8 min
Chambers et al. [13] Part 2	8	t	75% VO2max	6.4% MD versus PLA (artificially sweetened)	Time trial ~60 min (837 ± 68 kJ)	Yes	62.6 ± 4.7 min versus 64.6 ± 4.9 min
Beelen et al. [17]	14	t	75% Wmax	6.4% MD versus PLA (water)	Time trial ~60 min (1053 ± 48 kJ)	No	68.14 ± 1.14 min versus 67.52 ± 1.00 min
Whitham and Mckinney [18]	7	mt	65% VO2max	6% MD + 3% LJ versus 3% LJ PLA	Time trial ~45 min	No	9333 ± 988 m versus 9309 ± 993 m
Rollo et al. [11]	10	t	RPE = 15	6% CHO versus PLA (artificially sweetened)	Time trial ~30 min	Yes	6584 ± 520 m versus 6469 ± 515 m

t: trained, mt: moderately trained, MD: maltodextrin, PLA: placebo, CES: carbohydrate-electrolyte solution, LJ: lemon juice; RPE: Rate of perceived exertion.

Data are reported as means ± standard deviation.
